# Mesenchymal Stem Cells in Multiple Sclerosis: Recent Evidence from Pre-Clinical to Clinical Studies

**DOI:** 10.3390/ijms21228662

**Published:** 2020-11-17

**Authors:** Agnese Gugliandolo, Placido Bramanti, Emanuela Mazzon

**Affiliations:** IRCCS Centro Neurolesi “Bonino-Pulejo”, Via Provinciale Palermo, Contrada Casazza, 98124 Messina, Italy; agnese.gugliandolo@irccsme.it (A.G.); placido.bramanti@irccsme.it (P.B.)

**Keywords:** multiple sclerosis, mesenchymal stem cells, preclinical models, clinical trials

## Abstract

Multiple sclerosis (MS) is an autoimmune, demyelinating disease of the central nervous system. Nowadays, available therapies for MS can help to manage MS course and symptoms, but new therapeutic approaches are required. Stem cell therapy using mesenchymal stem cells (MSCs) appeared promising in different neurodegenerative conditions, thanks to their beneficial capacities, including the immunomodulation ability, and to their secretome. The secretome is represented by growth factors, cytokines, and extracellular vesicles (EVs) released by MSCs. In this review, we focused on studies performed on in vivo MS models involving the administration of MSCs and on clinical trials evaluating MSCs administration. Experimental models of MS evidenced that MSCs were able to reduce inflammatory cell infiltration and disease score. Moreover, MSCs engineered to express different genes, preconditioned with different compounds, differentiated or in combination with other compounds also exerted beneficial actions in MS models, in some cases also superior to native MSCs. Secretome, both conditioned medium and EVs, also showed protective effects in MS models and appeared promising to develop new approaches. Clinical trials highlighted the safety and feasibility of MSC administration and reported some improvements, but other trials using larger cohorts of patients are needed.

## 1. Introduction

Multiple sclerosis (MS) represents a chronic inflammatory, demyelinating, neurodegenerative disease of the central nervous system (CNS). The hallmark of the pathology is the accumulation of demyelinating lesions both in white and grey matters in the brain and spinal cord [1]. Clinically isolated syndrome (CIS) is indicated as the first clinical manifestation of the disease, showing features of inflammatory demyelination, but the MS criteria are not completely fulfilled. In the majority of patients, reversible episodes of neurological deficits, indicated as relapses, characterize the initial phases of the disease, that is indicated as relapsing remitting MS (RRMS). After, the development of permanent neurological deficits and the progression of clinical disability become prominent, indicating a secondary progressive MS (SPMS). Only a small number of patients has a progressive disease course since the onset, indicating a primary progressive MS (PPMS) [2]. RRMS shows an earlier onset, appearing typically between 20-35 years of age, while PPMS at about 40 years of age [1]. About three million people are affected by MS, and in particular, females are more affected than males [3].

MS is based on an autoimmune mechanism, and specifically the myelin antigens represent the targets. T lymphocytes, both CD4+ T cells and CD8+ T cells, take part in the pathological process, and in particular MS is triggered by pathogenic T helper (Th) 17, Th1, and CD8+ autoreactive T lymphocytes directed against myelin components. In addition, in the demyelinated areas, resident microglia and macrophages are also present [4]. Even if MS was for a long time considered as a T cell-mediated disease, the positive effects exerted by antibodies targeting CD20, highlighted the role of B cells in the immunopathogenesis of MS. In particular, B cells’ role in MS is not limited to the antibody production, but a main role is played by their antibody-independent functions, which are the antigen presentation to T cells and the modulation of T and myeloid cell function through the secretion of cytokines [5,6,7].

Nowadays, therapeutic approaches aim to treat acute attacks and to improve symptoms. Disease-modifying therapies can modulate the immune system, exerting anti-inflammatory activity and reducing the rate of relapses. They can stabilize, delay or, only in some cases, slightly improve disability [8].

New treatments are needed and stem cell therapy is arising as a new strategy. Different stem cells can be used, such as hematopoietic stem cells [9], but mesenchymal stem cells (MSCs) seem promising. In this review, we focused on the studies involving the use of MSCs or their derivatives in in vivo models of MS and in patients affected by MS. Moreover, we also discussed the feasibility of autologous MSCs therapy. In order to select the studies, we performed a PubMed search, using the keywords “mesenchymal stem cell” and “multiple sclerosis”, collecting the works published in the last five years that evaluated the efficacy or the safety of MSCs transplantation in MS models and in MS patients. We also considered the studies that compared MSCs obtained from MS patients with those of healthy controls, in order to compare their characteristics with the aim to evaluate whether MS patients derived MSCs showed equal therapeutic potential.

## 2. Mesenchymal Stem Cells

MSCs are non-hematopoietic adult stem cells with self-renewal ability, originating from the mesoderm, but possess a multilineage differentiation capacity. Indeed, MSCs can differentiate not only toward mesoderm lineages, such as chondrocytes, osteocytes, and adipocytes, but also toward ectodermic and endodermic cells [10]. MSCs were first isolated from the bone marrow, but they are also found in adipose tissue, umbilical cord, dental tissues, birth-derived tissues, and others [11]. According to the Mesenchymal and Tissue Stem Cell Committee of the International Society for Cellular Therapy, the minimal criteria to define human MSCs are: (1) their plastic-adherence in standard culture conditions; (2) the expression of the surface molecules CD105, CD73, and CD90, and the lack of CD45, CD34, CD14 or CD11b, CD79α or CD19, and HLA-DR; (3) their capacity to differentiate toward osteoblasts, adipocytes, and chondroblasts in vitro [12].

The therapeutic potential of MSCs is associated to their differentiation capacity and paracrine effects. Indeed, MSCs are able to secrete different growth and trophic factors, cytokines, microRNAs, which form the so called secretome. The secretome includes both the conditioned medium (CM) and the extracellular vesicles (EVs), which in turn can be distinguished in apoptotic bodies, microvesicles, and exosomes (Exo) [13,14,15].

In the context of treating MS, MSCs were shown to be able to differentiate toward oligodendrocytes, expressing oligodendrocyte progenitor cell (OPC) markers, such as A2B5 and oligodendrocyte transcription factor (Olig2). A smaller percentage of cells expressed also myelin basic protein (MBP), which is a marker of mature oligodendrocyte. A very low percentage of cells also expressed the astrocyte marker glial fibrillary acidic protein (GFAP) [16]. The CM derived from MSCs was also found to be able to enhance oligodendrogenesis in hippocampal neural stem cells and to support their integration into neuronal networks [17].

Another important characteristic of MSCs is their capacity to move towards the damaged area following chemical gradients. This property is known as homing [18].

MSCs possess anti-apoptotic, anti-inflammatory, and immunomodulatory properties. They take part in both innate and adaptive immunity. The immunomodulatory actions may be exerted by a direct contact with immune cells, or by MSCs paracrine activity. In particular, it is reported that MSCs inhibit Th17 and Th1 cell differentiation, while they promoted regulatory T (Treg) cell differentiation and influenced macrophage polarization inducing the formation of anti-inflammatory M2 macrophages instead of M1 inflammatory ones [19,20]. However, it is important to notice that the division of macrophages in M1 and M2 phenotypes was adopted in order to simplify data interpretation, but it is not accurate [21]. Indeed, different subgroups of macrophage activation states were proposed on the basis of transcriptome network analysis [22].

## 3. MSCs Administration in MS Experimental Models

The potential beneficial effects of MSCs obtained from different sources were evaluated in MS experimental models, with the aim to study the actions exerted by the different MSCs sources, the different routes of administration, the right number of cells that must be administered to obtain the therapeutic effects, and also the influence of the timing of administration.

Bone marrow MSCs (BM-MSCs) administration through the intraperitoneal route improved experimental autoimmune encephalomyelitis (EAE), as demonstrated by the amelioration of clinical score as well as the reduced inflammatory infiltration and demyelination in the spinal cord. Beneficial actions of BM-MSCs depended on their immunomodulatory properties. Indeed, they inhibited CD4+ and CD8+ T cell activation, induced Treg and modulated macrophage polarization from M1 to M2 phenotype, leading to a reduced production of pro-inflammatory cytokines [23]. In agreement, BM-MSCs intraperitoneal transplantation other than ameliorate clinical scores, reduced tumor necrosis factor (TNF)-α, myeloperoxidase (MPO), thiobarbituric acid reactive substances, and nitric oxide (NO) brain levels, while interleukin (IL)-10 and brain glutathione (GSH) levels increased. Interestingly, mice treated with methylprednisolone (MP), a glucocorticoid used for MS, also showed a milder disease, but MSC effects were precocious [24]. The signaling IL17/IL17 Receptor A (IL17RA) may mediate MSCs therapeutic actions in EAE. Indeed, mice that received IL17RA^−/−^ MSCs showed a more severe disease, while wildtype MSCs were able to decrease the clinical disease score. This evidence indicated that the expression of IL17RA was a requisite for MSCs in order to exert their protective functions [25].

The intravenous route of administration also appeared to be efficacious. Also in this case, BM-MSCs improved neurobehavioral outcomes and reduced inflammatory infiltration as well as demyelination in the spinal cord. BM-MSCs also alleviated blood-brain barrier (BBB) disruption, which is one of MS features, reducing IgG protein leakage and increasing tight junction proteins occludin and ZO-1. Moreover, the amelioration of BBB damage was suggested to be mediated by decreased aquaporin 4 (AQP4) and A2B adenosine receptor (A2BAR) levels in the spinal cords of EAE mice [26]. Interestingly, using non-invasive X-ray phase-contrast tomography, it was observed that BM-MSCs administration reduced vascular vessel alterations, myelin, and neuronal injuries in an EAE model [27]. In addition to the previously reported improvements, the systemic administration of BM-MSCs through the intravenous route exerted also beneficial actions on retinal ganglion cell function in an EAE model. This effect was present in association with an amelioration of motor-sensory impairment, pattern electroretinogram amplitude, and preserved retinal nerve fiber layer. Moreover, the transcriptomic analysis of retinas and optic nerves showed that BM-MSC transplantation reduced endoplasmic reticulum stress and hypoxia-inducible factor 1 (HIF-1) signaling, while it increased the expression of genes involved in neuroprotection and repair, influencing also cholesterol metabolism [28].

It is also important to establish the effects of BM-MSCs on the basis of the time of administration. In particular, it was evaluated if BM-MSCs were able to exert protective effects when they were administered at the onset, at the peak or after EAE stabilization. BM-MSCs administered at the onset of EAE improved clinical symptoms, while when BM-MSCs were injected at the peak of EAE they induced transient positive effects. On the contrary, no improvements were found when MSCs were transplanted after EAE stabilization. Moreover, EAE atypical signs, including unbalanced gaits or rotatory defects, were detected in animals that received MSCs at the peak or after EAE stabilization. MSCs transplantation at all times reduced T-box transcription factor TBX21 (T-bet), RAR-related orphan receptor γ (RORγT), and forkhead box P3 (Foxp3) mRNA levels in the brain of EAE mice, associated with Th1, Th17, and Treg, respectively, suggesting a reduction of these T cell populations. However, a higher proportion of Treg over Th1 and Th17 lymphocytes was present in mice that received MSCs at the disease onset. Higher levels of IL-6 were found at the disease onset, together with other cytokines associated with Th1 and Th17 lymphocytes. On the contrary, at the EAE peak only IL-6 plasma levels remained elevated, while the majority of the cytokines decreased. At the time of EAE stabilization, IL-6 decreased to levels similar to those at the onset of EAE. MSCs, independently of the administration time, were able to decrease IL-6 levels and this effect was lasting. The authors speculated that IL-6 reduction could restore BBB function, reducing as a consequence the infiltrating leukocytes [29].

Even if the majority of the studies evaluated the MSC efficacy in EAE models, their effect was also evaluated in the cuprizone model, a non-T cell mediated model. In contrast to the previous works, a report indicated that MSCs inhibited the activation and function of Th17 cells, not influencing Th1 activation. In the EAE model, MSC transplantation did not exert protective actions, showing a higher frequency of Th1 cells in the brains, while Th17 cells were not affected. On the contrary, in the cuprizone model, MSCs intraperitoneal administration showed a positive effect, increasing myelination [30]. In addition, BM-MSCs administration showed effects on microglia polarization. Specifically, MSCs treatment increased the M2 polarization, which produced anti-inflammatory cytokines, while decreasing the M1 phenotype, involved in the production of pro-inflammatory cytokines [31]. BM-MSCs were also able to reduce microgliosis and astrocytosis through the secretion of neuroprotective factors, such as transforming growth factor (TGF)-β and CX3CL1. In addition, remyelination was also induced. On the contrary, pro-inflammatory cytokines, including IL-1β and TNF-α, and chemokine levels decreased [32].

Multiple BM-MSCs intraperitoneal injections were also able to reduce demyelination in the corpus callosum and an increased expression of MBP was found. However, BM-MSCs were not detected inside the corpus callosum [33]. Instead, BM-MSCs administered intravenously were able to migrate and engraft in the brain, where they reduced demyelination and enhanced remyelination in the corpus callosum. It is important to notice that remyelination was not due to BM-MSCs differentiation into the oligodendroglial phenotype. BM-MSCs modulated also the glial response and reduced apoptosis. These data suggested that the therapeutic potential of BM-MSCs against MS did not depend only on their immunosuppressive and immunomodulatory capacity, but also on their ability to enhance endogenous repair [34].

In rats with induced demyelination, both BM-MSCs obtained from young and old rats were able to migrate into the lesions, but only those obtained from young rats induced differentiation of OPC. No transdifferentiation of BM-MSCs into oligodendrocytes was found. These results indicated that aging limited BM-MSCs beneficial capacity to induce oligodendrocyte differentiation and myelination [35].

Given that BM-MSCs were the first to be discovered, it is not surprising that a lot of studies used bone marrow as a source of MSCs. However, also other types of MSCs were tested for their efficacy against MS in experimental models. Murine adipose tissue-derived MSCs (AD-MSCs) were able to reduce disease severity when administered both at the onset or during the acute phase of the disease in EAE mice. Moreover, also inflammatory cell infiltration and demyelination were reduced and AD-MSCs administration prevented dendritic cell (DC) function. Interestingly, also human AD-MSCs, administered during the acute phase, were able to exert protective effects reducing disease score [36].

The beneficial effects of AD-MSCs were evaluated in dogs with neurological lesions caused by the distemper virus as MS model. During the first year after AD-MSCs administration, some improvements were found and myoclonus was reduced to a moderate grade in some animals. Interestingly, one year after AD-MSCs administration, all the dogs were able to move independently [37].

Moreover, obesity impaired the therapeutic effects of AD-MSCs, indeed AD-MSCs derived from obese people failed in improving EAE symptoms. AD-MSCs derived from obese people stimulated with IFN-γ showed higher expression of pro-inflammatory cytokines compared to AD-MSCs from non-obese donors. These results may indicate the unsuitability of AD-MSCs derived from obese donors [38].

The efficacy of AD-MSCs was compared to stromal vascular fraction (SVF) in an EAE mouse model. The SVF of adipose tissue includes, other than AD-MSCs, also adipocytes, leukocytes, endothelial cells, hematopoietic cells, and smooth muscle cells. It was obtained by a digestion of a harvested adipose tissue. Both SVF and AD-MSCs improved clinical score and motor function, reduced inflammatory cell infiltration and demyelination. However, SVF induced better improvements thanks to the modulation of some inflammatory factors; indeed a higher level of IL-10 was found compared to AD-MSCs treatment [39]. The effects of administration of SVF and AD-MSCs during the disease progression were also evaluated. SVF and AD-MSCs treatments temporarily increased disease severity. However, only EAE mice that received SVF cells showed improvements and a milder disease severity [40]. 

Dental tissues are arising as important sources of MSCs. Periodontal ligament stem cells (PDLSCs) were able to exert neuroprotective effects against EAE, ameliorating clinical score, reducing lymphocytic infiltration and demyelination. These effects were likely mediated by the increase of neurotrophic factors, such as nerve growth factor (NGF) and brain derived neurotrophic factor (BDNF). Furthermore, PDLSCs treatment reduced pro-inflammatory cytokines, CD4 cells, CD8α T cells, and apoptosis [41].

Also skin-derived MSCs (S-MSCs) delayed disease onset, reduced disease severity, demyelination and inflammatory cell infiltration in EAE mice. S-MSCs inhibited Th17 induction in spleen and CNS, decreasing as a consequence serum IL-17A levels together with interferon (IFN)-γ. In addition, the expression levels of Th2- and Treg-associated genes in splenocytes were increased. Altogether, the results indicated that S-MSCs inhibited Th1 and Th17 cell differentiation and induced Th2 and Treg cells *in vivo*, leading to improvements of EAE. In inflammatory conditions, S-MSCs released high levels of soluble TNF receptor 1 (sTNFR1), capable of binding and antagonizing TNF-α. It is important to notice that TNF-α can promote Th17 differentiation. Knocking down sTNFR1 in S-MSCs suppressed their inhibition of Th17 differentiation [42]. 

Wharton’s jelly-derived MSCs (WJ-MSCs) administration increased remyelination and oligodendrocyte number in a cuprizone MS model. These effects were associated with the reduction of astrogliosis and microgliosis in the corpus callosum. The positive effects were due to the inhibition of oxidative stress and mitochondrial dysfunction, as demonstrated by the reduction of malondialdehyde (MDA), while GSH and superoxide dismutase (SOD) increased together with the expression levels of mRNA involved in mitochondrial biogenesis [43]. It was reported that WJ-MSCs were able to inhibit proliferation of activated T cells through both direct contact and paracrine mechanisms. Indeed, MSCs released different immunomodulatory molecules, such as indoleamine 2,3-dioxygenase 1 (IDO1). When WJ-MSCs were administered at the onset or at the chronic phase of the disease, they were able to improve clinical score in EAE rats, reducing T cell proliferation. Interestingly, WJ-MSCs that were exposed to IFN-γ, IL-1β, and TNF-α were more efficacious in suppressing T cells. However, WJ-MSCs exposed to cytokines did not exert protective actions against EAE, maybe because of a rejection due to the enhanced immunogenicity [44]. 

Human umbilical cord MSCs (UCMSC) administration in cynomolgus monkeys with EAE improved MS clinical symptoms, reduced demyelination and inflammation. The protective effects were due to UCMSC modulation of Treg populations and natural killer (NK) cells, and to the suppression of astrocyte activation [45].

Also human amnion mesenchymal cells (AMCs) were able to reduce clinical score, inflammation, and to induce remyelination in an EAE model. Indeed, pro-inflammatory cytokine levels in spleen and CNS and CD4+ and CD8+ T cells in the CNS were reduced in EAE mice transplanted with AMCs. AMC treatment also induced the production of neurotrophic factors, such as NGF and BDNF in the CNS of EAE mice [46]. 

Placental derived MSCs (PMSCs) administered intravenously were found in the brain tissue. PMSCs transplantation decreased inflammation and axonal injury. While degenerating neurons decreased, oligodendrocyte precursors increased together with neurotrophic factors in the brain. However, rats receiving PMSCs at the onset of the disease showed better improvements compared to animals that received PMSCs at the peak of the disease [47]. Jiang et al., confirmed the protective actions of PMSCs, but also of embryonic MSCs (EMSCs), in an EAE model. Indeed, both cells decreased infiltrating inflammatory cells and gliosis. Consequently, pro-inflammatory cytokines and apoptosis were also reduced. MSCs transplantation also reduced demyelination, inhibited axon loss, and restored the levels of neurotrophins. All these effects induced an improvement of neurological functions. In addition, PMSCs and EMSCs could migrate into the inflamed tissues and expressed markers of neural and glial lineages [48]. EMSC in spheres intrathecally injected into the CNS of EAE monkeys, reduced clinical symptoms, brain lesion development, and demyelination. Interestingly, EMSCs were able to transdifferentiate toward neural cells in vivo [49]. 

Also, decidua derived MSCs (DMSCs), obtained from human placental extraembryonic membranes, were tested in an EAE model. DMSCs administration before disease onset resulted in a delay of EAE development. Moreover, DMSCs reduced demyelination and inflammatory cell infiltration. The treatment of animals that already presented moderate symptoms also resulted in a less severe EAE, with a reduction in scores. Interestingly, long-term treatment prolonged the therapeutic effects of DMSCs. Spleen cells of the mice treated with DMSCs showed a low proliferation in response to antigen, a decreased production of IL-17, and an increased production of the anti-inflammatory cytokines IL-4 and IL-10. Moreover, lower RORγT and higher GATA-3 expression levels, which control the definition of Th17 and Th2 phenotypes respectively, were detected in DMSC-treated mice [50]. 

In contrast to the previous studies, which highlighted the beneficial effects of MSCs in experimental MS models, one piece of research work found that BM-MSCs did not ameliorate EAE and no MSC migration into the CNS was detected. However, they were found in liver, lung, and spleen [51]. In line with this study, human, murine, canine MSCs transplanted intraventricularly into the cerebrospinal fluid or into the lesion at the onset of OPC proliferation or at the peak of OPC proliferation were not able to exert regenerative effects in a cuprizone model. It was supposed that MSC may not be able to exert proregenerative actions in a model where peripheral immune cells were not involved, suggesting that the peripheral immune system is needed to obtain MSC positive effects [52].

The efficacy of mouse multipotent adult progenitor cells (mMAPCs) was compared to BM-MSCs in an EAE model. They indicated that mMAPCs showed more prominent effects compared to MSCs. In particular, mMAPCs reduced clinical and inflammatory scores in EAE mice and increased the expression of genes encoding for trophic factors, such as BDNF, Neurotrophin-3 (NT-3), GAP43, PDGFR, nestin in the spinal cord, indicating major neuroprotective and regenerative effects of mMAPC [53]. 

Interestingly, it was found that BM-MSCs obtained from EAE mice did not improve EAE in comparison to naïve MSCs. The same results were obtained with CM derived from MSCs isolated from EAE animals. These results may be explained taking into account that EAE-MSCs released higher levels of pro-inflammatory cytokines compared to naïve MSCs and possessed less differentiation potential. Also, human BM-MSCs derived from MS patients showed less therapeutic effects compared to naïve MSCs in treating EAE and secreted higher levels of pro-inflammatory cytokines [54]. 

**Figure 1:**
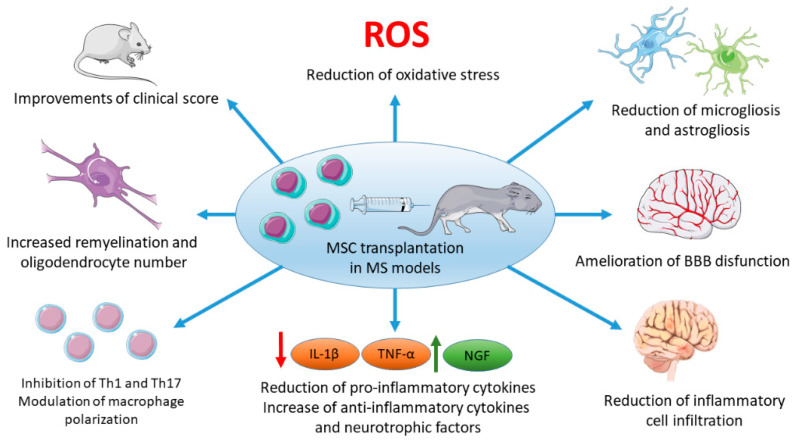
Beneficial effects of mesenchymal stem cells (MSCs) in multiple sclerosis (MS) experimental models. The figure was created using the images from Servier Medical Art [55], licensed under a Creative Commons Attribution 3.0 Unported License [56]. Abbreviations: BBB, blood-brain barrier; NGF, nerve growth factor.

The potential beneficial effects of MSCs on MS models are summarized in Figure 1.

The majority of the results indicated that MSCs were able to exert protective effects in MS experimental models, reducing inflammatory cell infiltration, disease score, demyelination, and BBB disruption, thanks to their immunomodulatory function. The beneficial effects of MSCs were not dependent on the sources. Indeed, the majority of the works used BM-MSCs, but also the other MSCs types exerted protective effects. It is not easy to determine the best MSC source, because the reviewed works did not compare the different MSCs. BM-MSCs were the first to be discovered and isolated, but nowadays other sources may present the advantage of an easier isolation method, such as AD-MSCs or dental MSCs. Interestingly, both human or animal derived MSCs exerted the protective effects, suggesting that at least in vivo, xenogeneic MSC transplantation is also efficacious. The number of MSCs administered was in the range from 3 × 10^5^ to 1 × 10^7^, except for a work on monkeys where 2 × 10^7^ cells were administered. These doses exerted protective effects both when administered alone or in multiple injections. Interestingly, the lowest dose was administered directly into the lateral ventricle, while higher doses were often administered through intraperitoneal or intravenous injections. This may suggest that, although all the administration routes seem to be valid, the systemic administration may require higher doses of cells. It is important to notice that MSCs were able to exert protective effects particularly when administered at the onset of clinical symptoms, but not after MS stabilization. 

MSCs may present also long-term effects, as reported in [37], where dogs were able to move one year after AD-MSCs administration, or in [29], where BM-MSCs administered at the onset of EAE showed lasting effects on the clinical score. Moreover, multiple injections were associated with prolonged effects. 

It is important to notice that the reviewed studies used animals of only one sex. However, gender differences exist in EAE. It is becoming evident that sex-dependent activation pattern of immune cells may exist in the injured CNS during EAE. In particular, engraftment of microglia-like cells after microglia depletion enhanced EAE in female mice, but not in male [57]. It also seems that hypersensitivity in female EAE mice may be more immune-driven, while pain in EAE male mice may depend on neurodegenerative and plasticity-related processes [58]. A different susceptibility to EAE may exist also between the different strains. However, no sex differences were noted in the C57BL/6 mice [59], which represents the most used mouse strain. However, the results in the reviewed papers indicated that both male and female animals showed improvements in EAE.

An overview of the studies described in this paragraph is shown in Table 1.

### 3.1. Use of Differentiated MSCs in Multiple Sclerosis Experimental Models

Some studies evaluated the effects of differentiated MSCs in MS experimental models. Indeed, the differentiation may increase the potential benefits of MSCs.

The therapeutic potential of neuralized MSCs (NMSCs), transdifferentiated into neuronal spheres, and differentiated NMSCs, where NMSCs were differentiated into functional neuronal cells, was evaluated. Administration of NMSCs and differentiated NMSCs at the onset of the chronic phase of EAE ameliorated disease score compared to controls and to naïve MSC. NMSCs and especially differentiated NMSCs induced a reduction in inflammation, axonal loss, and demyelination. Therefore, NMSCs and differentiated NMSCs may represent a better option for cell-based therapy compared to naïve MSC [60].

MSCs-derived neural progenitors (MSCs-NPs) exhibited greater therapeutic potential, suppressing the proliferation of pathogenic myelin oligodendrocyte glycoprotein (MOG)-specific T cells, decreasing IFN-γ production and increasing anti-inflammatory IL-10 production. These results may be explained taking into account that MSCs-NPs produced higher levels of PGE2 in vitro compared to MSCs. MSCs and MSCs-NPs treatments decreased EAE clinical scores, but MSCs-NPs exerted a better effect [61]. Also, umbilical cord blood mesenchymal stromal cells (UCB-MSC) derived neural progenitor cells in EAE improved clinical score and reduced leukocyte infiltration [62].

BM-MSCs differentiated toward OPCs, transplanted in a model of local demyelination, resulted in cell differentiation into mature oligodendrocytes positive for MBP. The OPC administration reduced demyelination and increased remyelination [63]. 

The results indicated that MSCs differentiated toward neuronal progenitors or OPCs exerted positive effects against EAE, and also superior effects compared to control MSCs. The main effects were a reduction of inflammation and demyelination. Moreover, the derived OPCs were able to differentiate toward mature oligodendrocytes. However, different methods were used to differentiate cells. The number of administered cells was in the range 2 × 10^5^–1 × 10^6^. Also in this case, mainly BM-MSCs were used and both human or murine MSCs were able to exert protective effects. 

An overview of the studies described in this paragraph is shown in Table 2.

### 3.2. Use of Preconditioned MSCs in Multiple Sclerosis Experimental Models

When MSCs are transplanted they may have a short survival caused by the oxidative stress and inflammation at the injury site. For this reason, strategies that improve MSC survival and therapeutic effects are needed. The preconditioning of MSCs with natural phytochemicals or with different compounds was shown to influence MSCs characteristics, including survival and may improve therapeutic potential.

It was suggested that IFN-γ may improve the immunosuppressive ability of MSCs, probably through the upregulation of IDO. IFN-γ preconditioned UCMSCs improved body weight loss and clinical symptoms compared with naïve UCMSCs and control groups. IFN-γ-preconditioned UCMSCs administration also decreased serum IL-17A and TNF-α levels. The improved therapeutic potential may be due to the increase in IDO1 levels in UCMSCs preconditioned with IFN-γ [64].

Also, natural compounds were tested to evaluate if they were able to improve MSCs features. Tetramethylpyrazine (TMP) is obtained from the traditional Chinese herb *chuan xiong*. It possesses anti-inflammatory, anti-oxidant, and anti-apoptotic capacities. TMP was able to increase cell survival and to exert antioxidant actions in UCMSCs treated with hydrogen peroxide. Moreover, TMP-preconditioned UCMSCs improved EAE, decreasing clinical score, inflammatory cell infiltration, NLRP3 levels, demyelination, and BBB disruption. TMP-preconditioned UCMSCs exerted better effects compared to non-treated cells [65].

Pretreatment with stromal cell-derived factor 1α (SDF-1α), a member of CXC cytokines, may enhance chemotaxis and homing to the damaged site. The preconditioning with SDF-1α increased C-X-C chemokine receptor type 4 (CXCR4) expression on the BM-MSCs surface and ameliorated BM-MSCs survival in vitro. The intranasal delivery of SDF-1α preconditioned BM-MSCs was shown to improve remyelination in a cuprizone model. Transplantation of SDF-1α-preconditioned BM-MSCs improved CXCL12 level in the brain, spatial learning, memory, and myelination. Moreover, preconditioning of BM-MSCs with SDF-1α increased the number of cells that reach the brain and reduced the protein levels of GFAP and Iba-1. On the contrary, the expressions of the marker of oligodendrocyte immaturity Olig-2 and the marker of oligodendrocyte maturity adenomatous polyposis coli (APC) increased [66]. 

Given that 17 β-estradiol (17β-ED) administration was reported to ameliorate EAE, and that pregnancy decreased MS clinical symptoms, the effects of 17β-ED preconditioning were evaluated in an EAE model. EAE rats treated with MSCs preconditioned with 17β-ED showed improvements in clinical score and neuropathological changes. Moreover, a reduction in serum MPO and NO was observed in EAE animals treated with 17β-ED primed MSCs compared to untreated MSCs. Also the reduction of IL-17 and TNF-α in stimulated splenocyte ex vivo was found [67]. Moreover, lymphocyte infiltration into the spleen decreased. Gene expression of the pro-inflammatory cytokines IL-17, TNF-α, and IFN-γ, and the matrix metalloproteinases (MMP) 8 and MMP9 decreased, while the levels of the anti-inflammatory cytokines IL-10, IL-4, and TGF-β increased. Thanks to these immunomodulatory properties, rats treated with MSCs preconditioned with 17β-ED showed a reduced disease severity. Then, the results indicated that 17β-ED enhanced the efficacy of MSCs transplantation [68].

Laser irradiation can activate the cells and induce cell proliferation. Interestingly, laser-activated non-expanded SVF in an MS dog model improved clinical signs and remyelination. Moreover, migration of the injected cells into the lesion was found [69].

These results indicated that pre-treatment may be an interesting approach to enhance MSCs’ properties. Pretreatments were able to increase cell survival, proliferation or homing capacity, properties useful in the context of stem cell therapy. Regardless of the administration route and of the MSCs source, MSCs transplantation exerted protective effects.

An overview of the studies described in this paragraph is shown in Table 3.

### 3.3. Use of Engineered MSCs in Multiple Sclerosis Experimental Models

Given that IFN-β is commonly used as a disease modifying drug for the treatment of RRMS, the effects of genetically modified AD-MSCs expressing murine IFN-β on the EAE model were evaluated. Engineered MSCs and control MSCs decreased inflammatory cell infiltration. Moreover, IFN-β expressing MSCs were more efficacious compared to control MSCs, and their administration-induced Tregs and IL-10 production, while reduced IL-17 levels [70]. Another study demonstrated that the transduction of IFN-β to AD-MSCs maintained and even enhanced the properties of AD-MSCs. In particular, autologous transplantation of IFN-β-AD-MSCs in a RR-EAE model reduced disease score, activated microglia and inflammatory cell infiltration. The allogeneic transplantation of IFN-β-AD-MSCs in a chronic progressive EAE model also reduced disease score, demyelination, and infiltration in spinal cords. Transduced MSCs were more efficacious than control ones [71].

The effects of the administration of MSCs with triple P-selectin glycoprotein ligand-1 (PSGL1)/Sialyl-Lewis^x^ (SLeX)/IL-10 engineering in a mouse EAE model were evaluated. PSGL-1/SLeX transfection improved MSC homing into the spinal cord of EAE mice. MSCs engineered with PSGL-1/SLeX/IL-10 showed better therapeutic properties compared to control MSCs, ameliorating clinical score, myelination, and decreasing inflammatory infiltration into the spinal cord of EAE mice [72].

UCMSCs transfected with sphingosine kinase 1 (SPK1) gene were more efficacious compared to the control UCMSCs, and in particular they reduced neurological deficits, inflammatory cell infiltration, demyelination, and astrogliosis. In addition, the administration of engineered MSCs reduced pro-inflammatory cytokines in the serum of EAE mice. Moreover, the treatment also inhibited NK development in EAE spleen, while it increased the Treg cells [73]. 

The transfection of MSCs in order to induce the expression of specific genes also increased their therapeutic potential, ameliorating anti-inflammatory properties, homing, and myelination. The presented works demonstrated that the transfected MSCs showed superior effects compared to the control cells. Interestingly, all the works used the intravenous route of administration.

An overview of the studies described in this paragraph is shown in Table 4.

## 4. MSCs Secretome in Multiple Sclerosis Experimental Models

The use of secretome may present some advantages in terms of manufacturing, storage, and shelf life. Indeed, the administration of MSCs requires MSCs expansion in vitro in order to obtain the dose useful for transplantation. Then, the administration of secretome is easier, more economical, and faster. Moreover, some studies indicated the poor survival of MSCs after injection, indicating that the main therapeutic properties may depend on their secreted molecules. The treatment with the secretome instead of MSCs also resolves several safety problems, such as immunocompatibility and tumorigenicity. 

CM obtained from BM-MSCs was administered through the nasal route in an EAE model induced by the administration of MOG or recombinant MOG (rMOG). Using rMOG, B lymphocytes were fully activated and played a main role in the pathology. Interestingly, CM administration was able to delay disease onset and to reduce disease severity in mice with rMOG-induced EAE, but not in those with MOG-induced EAE. Moreover, CM administration in rMOG EAE mice reduced demyelination, B cells infiltration, and microglia activation in the CNS, as well as the IgG and IgM levels in serum and pro-inflammatory cytokines in blood. Interestingly, the nasal route of administration showed the same therapeutic benefits of intraperitoneal and intravenous administrations. Then, nasal route represents a non-invasive administration, that bypassing the BBB, allows a direct access to the brain [74].

The CM obtained from stem cells from human exfoliated deciduous teeth (SHED) was also evaluated for its protective effects in EAE mice. Interestingly, SHED-CM ameliorated clinical scores, decreased demyelination, axonal injury, inflammatory infiltrates, as well as the expression of pro-inflammatory cytokines in the spinal cord. In addition, SHED-CM induced a shift from M1 to M2 macrophage phenotypes. SHED-CM was also able to suppress proliferation of MOG–specific CD4+ T cells and their production of pro-inflammatory cytokines in vitro. One of the main compounds found in SHED-CM was the secreted ectodomain of sialic acid–binding Ig-like lectin-9 (ED–Siglec-9). Interestingly, the administration of ED–Siglec-9 to EAE mice showed similar effects to SHED-CM treatment. On the contrary, the CM depleted of ED–Siglec-9 was not able to exert protective effects. These results indicated that ED–Siglec-9 represented a main factor responsible for the protective effects exerted by SHED-CM in EAE. On the contrary, Hepatocyte growth factor (HGF) depletion did not suppress CM protective effects, suggesting that HGF had little effect on the efficacy of SHED-CM [75]. 

Interestingly, AD-MSCs or their CM improved clinical scores and body weight of EAE mice in the same way. However, the administration of CM reduced inflammatory infiltration more efficiently than AD-MSCs. CM also suppressed splenocytes proliferation in response to different antigens. In addition, IFN-γ and IL17 were reduced in MOG-treated splenocytes from CM and AD-MSC, and the reduction was more pronounced after treatment with CM. On the contrary, IL4 levels and Treg were particularly increased in MOG-treated splenocytes from AD-MSC group compared to CM. In vitro, the percentage of Tregs increased in cell to cell contact with MSCs and with their CM. However, more Tregs were differentiated in cell–cell contact of T cells with MSCs compared with CM [76].

Our group in a previous study evaluated the protective effects of CM obtained from human PDLSCs under hypoxia (H-PDLSCs-CM) in an EAE model. H-PDLSCs-CM treatment reduced clinical score, inflammatory cell infiltration, and demyelination. Interestingly, elevated levels of IL-37 were found together with the reduction of pro-inflammatory cytokines in EAE mice treated with H-PDLSCs-CM. Moreover, the treatment with H-PDLSCs-CM increased BDNF levels while it reduced oxidative stress, apoptosis, and modulated autophagy [77]. Our group had also suggested that the immunosuppressive role of CM and Exo/microvesicles (EMVs) derived from PDLSCs of RRMS patients in an EAE model was at least in part due to the presence of soluble immunomodulatory factors, NALP3 inflammasome inactivation, and NF-κB reduction. At first it was found that the treatment with CM and EMVs reduced clinical score and inflammatory cell infiltration. Moreover, administration of CM and EMVs derived from PDLSCs obtained from RRMS patients caused a reduction of the inflammasome components NALP3, Cleaved Caspase 1, IL-1β, and IL-18 levels, together with Toll-like receptor-4 and NF-κB. Interestingly, CM obtained from both healthy and RRMS subjects contained the anti-inflammatory cytokines IL-10, TGF-β, and SDF-1α, and to a lesser extent IL-15, MCP-1, and MIP-1α [78]. The CM derived from WJ-MSCs differentiated toward oligodendrocytes was able to attenuate inflammatory cell infiltration and the expression of proinflammatory markers, while it improved remyelination and neurological scores in EAE mice. This CM was found to contain BDNF, glial cell-derived neurotrophic factor (GDNF), and ciliary neurotrophic factor (CNTF) [79]. 

The effects of EVs derived from AD-MSCs were evaluated in a Theiler’s murine encephalomyelitis virus (TMEV)-induced demyelinating disease used as a model of progressive MS. EVs administration in mice infected with TMEV improved motor deficits, reduced brain atrophy, increased cell proliferation in the subventricular zone, and decreased inflammatory infiltration in the spinal cord. EVs reduced neuroinflammation, indeed both GFAP and Iba-1 were reduced in the brain. On the contrary, myelin protein expression increased. In addition, EVs also modulate the activation of microglia, as evidenced by the changes in morphology. In addition, EVs treatment was also able to decrease plasma cytokine levels, mainly in the Th1 and Th17 phenotypes [80]. The effects of EVs derived from AD-MSCs were also tested in an EAE model. The treatments with EVs and AD-MSCs decreased clinical score and MOG-induced proliferation of splenocytes compared with untreated EAE control mice. In addition, inflammatory infiltrates and demyelination areas were reduced in animals treated with EVs or AD-MSCs [81]. 

High doses of EVs derived from PMSCs as well as PMSCs administered at the peak of the disease in EAE animals improved motor function. On the contrary, the lowest dose was not efficacious. For this reason, only the high EVs dose was further analyzed. Both PMSCs and PMSC-EVs were able to protect oligodendrocytes and increased myelination in the spinal cord. The results suggested that PMSCs effects were mediated by EVs. Evidence in vitro suggested that EVs-induced myelin regeneration through the induction of oligodendrocyte differentiation [82]. 

Intravenous injection of Exo, in native condition or obtained by MSCs stimulated by IFNγ (IFNγ-Exo), reduced the clinical score of EAE mice, reduced demyelination, decreased neuroinflammation, and upregulated the number of Tregs in the EAE spinal cords. Exo were mostly found in the liver and spleen of healthy and EAE mice. RNA sequencing evidenced that IFNγ-Exo contained anti-inflammatory RNAs. Moreover, they expressed anti-inflammatory and neuroprotective proteins [83].

Also, BM-MSC-derived Exo ameliorated inflammation and demyelination in an EAE model through the regulation of microglia polarization. Specifically, Exo increased M2-related markers and cytokine levels while M1-related markers and cytokines were reduced. In association, Exo treatment ameliorated behavioral scores, and reduced inflammatory cell infiltration and demyelination in a dose-dependent manner. Interestingly, the Exo proteomic analysis found various immune response-, inflammatory response-, and myelination-related proteins [84].

Hosseini Shamili et al. conjugated the amine groups on the Exo surface to the carboxylic acid-functionalized LJM-3064 aptamer, that has affinity for myelin and was demonstrated to induce remyelination. The construct Exo with aptamer reduced disease severity and in the prophylactic model it delayed disease manifestations. It was able to reduce inflammation and demyelination, reducing disease severity grade [85].

The results of these studies evidenced the great potential of MSCs-derived secretome in the treatment of MS. Both CM and EVs were able to induce protective effects. Moreover, the different routes of administration seem all valid and efficacious. These works evidenced that, at least in some cases, MSCs effects were mediated by their secretome and molecules important for secretome effects, such as ED–Siglec-9, were identified. Moreover, CM obtained culturing MSCs in hypoxia or in differentiation-inducing conditions were also efficacious. 

An overview of the studies described in this paragraph is shown in Table 5.

## 5. Combined Therapy Using MSCs in Multiple Sclerosis Experimental Models

The potential therapeutic effects of the combination of MP and human BM-MSCs were evaluated in an EAE model. The combined administration improved the clinical symptoms, reducing inflammatory cell infiltration and demyelination and increasing remyelination, compared to MP or BM-MSCs treatments alone. In addition, the combination reduced the levels of pro-inflammatory cytokines and increased anti-inflammatory cytokines in supernatants from MOG-reactivated splenocyte cultures. Moreover, analysis of MOG-reactivated T cells in spleen showed that the combination treatment decreased CD4+CD45+ and CD8+ T cells, and increased the number of Treg cells. The combination treatment increased apoptosis in MOG-reactivated CD4+ T cells [86]. Another study evaluated the combined treatment with MP and MSCs engineered to produce IFNβ in EAE mice. The combination reduced clinical score, inflammatory infiltration, BBB disruption, and increased remyelination. Moreover, the combination of MP and MSCs-IFNβ showed stronger immunomodulatory effects, reducing proinflammatory cytokines, and increasing anti-inflammatory cytokines [87]. 

Also, BM-MSCs and resveratrol delayed clinical symptom onset, reduced clinical scores, and inflammatory cell infiltration more efficiently than the single treatments. The combined treatment with BM-MSCs and resveratrol reduced serum proinflammatory cytokines and increased anti-inflammatory cytokines [88].

The combination of MSCs and nicotine reduced clinical score, neuropathological features, and disease disability, exerting a better result compared to MSCs or nicotine alone. The combination also reduced the pro-inflammatory cytokines IL-17 and TNF-α in the splenocytes, while IL-10 increased [89].

In another study it was found that adding rapamycin to BM-MSCs transplantation in EAE mice significantly reduced clinical score compared to the administration of BM-MSCs alone. The combined administration also decreased inflammatory cell infiltration and demyelination. Moreover, CD4 lymphocytes from EAE mice treated with BM-MSCs and rapamycin showed a lower proliferation response. Splenocytes of EAE mice treated with BM-MSC and rapamycin showed a lower ratio of Th1/Th2 [90].

Also the combination of Fasudil and BM-MSCs reduced clinical score, demyelination, and inflammatory cell infiltration compared to the single treatments. Moreover, the combination of Fasudil inhibited MSC-induced inflammatory signaling TLR-4/MyD88 and the inflammatory molecules IFN-γ, IL-1β, and TNF-α, but the combination did not contribute to the polarization of M1 to M2 phenotype. In addition, MSCs combination with Fasudil increased the expression of GDNF and BDNF compared with that of Fasudil [91]. 

Liu et al. preinduced MSCs with NT-3 and retinoic acid (RA) (NR-MSCs). Electroacupuncture (EA) increased NT-3 levels and promoted differentiation of NR-MSCs towards oligodendrocyte-like cells in the demyelinated spinal cord. NR-MSCs in combination with EA reduced demyelination and promoted remyelination. Also, the conduction of cortical motor-evoked potentials improved compared to controls [92]. 

The effects of microglial ablation, using PLX3397 that inhibits the colony stimulating factor 1 receptor, needed for microglia survival, and MSCs transplantation were evaluated in the cuprizone model. Treatments with PLX3397 and MSCs reduced microglia and astrocytes, respectively. The reduction of microglia was due to the CX3CL1/CX3CR1 axis. In association, an increase in oligodendrocytes was observed together with elevated levels of remyelination. As a consequence, also neurobehavioral deficits were recovered, such as motor coordination [93].

The combined therapy with MSCs also appeared promising. Indeed, it is possible to observe a synergistic effect of the combination, so that the animals treated with the combined therapy had advantages compared to animals treated only with MSCs or only with the different compounds. Interestingly, different substances were used in combination with MSCs: already known drugs, such as MP; natural compounds, including resveratrol; but also other treatments, such as EA. Also in this case, as already seen in the previous paragraphs, human or mouse/rat MSCs were used.

An overview of the studies described in this paragraph is shown in Table 6. 

## 6. Clinical Trials Using MSCs

Twenty-nine clinical trials were registered on Clinicaltrials.gov [94] and they were phase 1 or phase 2 trials. In particular, the available clinical trials had as their main aim to evaluate the safety of MSCs transplantation, but some studies also indicated some improvements. However, to really understand the therapeutic potential of MSCs therapy, larger cohorts of patients are needed. Here, we collected the published works regarding MSCs administration to MS patients. We selected not only the manuscript regarding registered clinical trials, but also trials approved by local ethical committees, published in the last five years.

An open-label, phase 1 trial confirmed the feasibility and safety of autologous BM-MSC intravenous transplantation in RRMS and SPMS patients. No severe adverse events or evidence of disease activation were recorded at a 6-month follow-up [95]. The same group indicated that MSC culture expansion was successful. The dose needed for transplantation was achieved in 16–62 days. The differences in growth rate were not dependent on demographic or MS features. Interestingly, the cytogenetic analysis evidenced changes on one chromosome in a control after prolonged in vitro culture [96].

Another open-label prospective phase I/IIa clinical study confirmed the feasibility and safety of autologous intrathecal BM-MSCs administration followed by CM in SPMS and RRMS patients, who failed the conventional therapies. No severe adverse events were recorded during the one year-follow up, but only minor ones, such as injection site reactions, including bruising, pain, and swelling, but also fever and headache. Constipation and tremor were also recorded, in one patient each. None of the patients experienced meningitis, encephalopathy, seizure, or allergic reactions. Interestingly, a trend for improvements in some tests was found, associated with the decrease of 4 and 3.5 points on the Expanded Disability Status Scale (EDSS) in two patients with SPMS. Moreover, IL-6, IL-8, VEGF, and MCP-1 were the cytokines found in elevated concentration in the CM. Interestingly, the highest secretion of IL-6, IL-8, and VEGF was found in the CM obtained from patients that showed the lowest number of white matter lesions at baseline [97]. Even if only a trend for an improvement of EDSS score was recorded in this study, and a larger cohort of patients is needed to evaluate the efficacy of MSC transplantation, this result is particularly encouraging because these patients failed the treatment with conventional therapies. 

The triple-blind, placebo-controlled study evidenced the safety of autologous AD-MSCs intravenous transplantation in SPMS patients. The main adverse events reported were urinary infections, respiratory infections, and anemia. The serious adverse events recorded were considered not related to MSCs treatment. However, no efficacy was found and larger cohorts of patients are needed to evaluate the therapeutic effects. It is important to notice that 4 patients were not treated because karyotype abnormalities in the cell products were found [98]. 

Also the intravenous administration of UCMSC was safe and feasible as shown by an open-label, single-arm, single-center phase ½ study. Indeed, after the administration of seven doses, no serious adverse events were recorded, but only moderate to mild ones, such as headache and fatigue, classified as probably related to the treatment. An amelioration of symptoms was found 1 month after the administration and in some cases also after 1 year. The improvements were found in EDSS scores, bladder, bowel, and sexual dysfunctions, in non-dominant hand average scores, in walk times, general perspective of a positive health change and improved quality of life. Magnetic resonance imaging (MRI) of the brain and the cervical spinal cord showed inactive lesions in 15/18 subjects after 1 year, and in particular, a patient showed nearly a complete resolution of brain plaques. However, it is important to note that 25% of patients did not receive other MS medications, while the others did not stop their usual therapy. This aspect may have a confounding effect; however, after MSCs transplantation, 20% of the patients reduced the intake of the medication [99].

Another trial evaluated the long-term safety and feasibility of UC-MSCs administration through the combination of intravenous and intrathecal routes in patients with RRMS. During the 10-year follow-up, no severe adverse events, including tumor formation or peripheral organ/tissue disorders, were recorded. Therefore, the combined intravenous and intrathecal UC-MSCs administration seemed to be safe and feasible [100].

A pilot study evaluated the safety of the intrathecal administration of MSC-NP in progressive MS patients. The patients received 2–5 intrathecal injections of escalating doses of autologous MSC-NPs and after patients were followed for an average of 7.4 years. The multiple dose treatment was well tolerated and no serious adverse events were recorded, only fever and headache. A group of patients also showed clinical improvements as suggested by the EDSS score. Some patients reported improvements of bladder and bowel functions. MRI evidenced that only one patient had disease progression since the last MSC-NP administration. On the contrary, the other patients showed no disease progression or abnormalities in MRI. However, improvements were only recorded when patients received more than 2 × 10^6^ cells [101]. The previous study was used to establish the dose for the following clinical trial. The open-label, single-arm, phase I clinical trial showed that the administration of 3 doses of MSC-NPs intrathecally was safe and well tolerated with no serious adverse events recorded. The minor adverse events found were fever, urinary tract infections, and headache. Moreover, MRI scans indicated no new lesions. Median EDSS score improved, especially in SPMS and ambulatory patients. Also, improvements in muscle strength and bladder function were reported. The trends recorded suggested that the therapeutic response may be more evident in ambulatory patients with SPMS, in contrast to non-ambulatory or PPMS patients [102]. An overview of the registered clinical trials is shown in Table 7.

Serum neurofilament light chain concentration is arising as a new biomarker of axonal injury in MS. In particular, Baldassari et al. found that its levels were higher in MS patients compared to control at baseline and no significant correlations were found between neurofilament light chain and clinical disability. A significant correlation between serum neurofilament light chain concentration and MRI measures of disease activity was found. However, after MSC administration, neurofilament light chain decreased but not in a statistically significant manner. Moreover, looking at each patient individually, some showed a decreased level, some showed an increase, while others presented no changes comparing the levels before and after the treatment. Therefore, MSC effects on neurofilament light chain were not clear [103].

Feng et al. evaluated MRI measures after MSC transplantation in MS patients. Lesional radial diffusivity (RD) and axial diffusivity (AD) were reduced before transplantation, and no modifications were found after. Mixed trends were found in normal-appearing white matter RD and AD before and after MSC administration [104]. 

In another study, MSCs were injected intrathecally in RRMS and SPMS patients who did not respond to disease modifying therapy. No severe adverse events were found. The patients reported fever and headache mainly related to lumbar puncture. SPMS patients did not show disease progression after the administration and some improvements were observed in the disability grade. MRI showed no new plaques or enhanced plaques [105].

Another study evaluated the efficacy of UCMSC intravenous transplantation of 7 doses, evidencing an improvement in symptoms. The patients showed adverse reactions, but not severe. The main adverse reactions were dizziness, headache, and fever. MRI evidenced a reduced number of foci and some improvements in symptoms were recorded [106]. 

Adipose-derived SVF was injected intracerebroventricularly in MS patients and all were stable or showed improvements [107]. Also, SVF infused intrathecally was shown to be safe [108]. An overview of these clinical trials is shown in Table 8. 

As reported by these studies, the major adverse effects reported after MSC transplantation were fever, headache, and some infections. Some of these adverse effects were associated with the administration, such as the adverse effects associated to the lumbar puncture or the pain at the injection site in case of intravenous administration. 

The results indicated that MSCs may have some therapeutic effects, that need to be confirmed in proper clinical trials. In particular, it is not easy to compare the effects of MSC transplantation with those obtained from the other approved therapies for MS. According to the reported trials, some patients were able to reduce the drug doses, and some data may suggest that MSCs transplantation may have effects also in patients not responding to disease modifying therapies. However, whether MSCs administration exert similar or superior effects compared to disease modifying therapies needs to be assessed.

The majority of the trials were performed on SPMS patients; however, it seems that SPMS may have major advantages compared to PPMS.

It is important to notice that also after prolonged follow-up, MSCs transplantation was shown to be safe, and beneficial effects appeared to be lasting, not limited at the time of administration.

The comparison of the different routes of administration is not easy. Indeed, it is necessary to take into account that each study used different cohort of patients, different cell types, different cell number. For this reason, a complete comparison is not possible. However, a meta-analysis indicated that intrathecal injection was associated with lower disability progression rate [109]. The same meta-analysis evidenced the safety of MSCs transplantation, given that temporary and mild adverse effects, such as fever, headache, backache, nausea/vomiting, iatrogenic meningitis, and urinary/respiratory infection were recorded.

## 7. Autologous Therapy: Is It Possible in MS?

Clinical trials evidenced the safety of autologous, but also heterologous administration of MSCs. Autologous MSCs transplantation presents some advantages; however, at first it is necessary to understand if MSCs obtained from MS patients showed the same characteristics of those isolated from healthy controls. 

Our group compared the stemness properties of PDLSCs obtained from healthy or RRMS subjects at passage 2 and 15. PDLSCs obtained from healthy donors and patients presented a similar expression of surface markers and cell proliferation rate. In addition, the differentiation capacities toward osteogenic, adipogenic, chondrogenic, and neurogenic lineage were also similar for PDLSCs derived from healthy subjects and RRMS patients. Both cells maintained the stemness properties even at the late 15th passage, while the senescence markers p16 and p21 showed increased expression at passage 15. These results indicated that PDLSCs obtained from healthy controls and MS patients showed similar features [110]. These findings may indicate the possibility of autologous stem cell therapy in MS patients. 

Contrary to this, another report indicated that MSCs isolated from MS patients showed a reduced in vitro expansion potential, accelerated senescence, and displayed accelerated shortening of telomere terminal restriction fragments in vitro [111]. 

It was found that MSCs obtained by MS patients exhibited a different transcriptional pattern, and less immunosuppressive functions compared to healthy donor MSCs [112]. MSCs obtained from MS patients showed a different susceptibility to nitrosative stress with enhanced senescence, reduction of SOD1, GSTP, Nrf2 and peroxisome proliferator-activated receptor gamma coactivator 1-α (PGC1α). These results indicated that MSC derived from MS patients showed a dysregulation of antioxidant responses [113].

Moreover, in a model of in vitro neurotoxicity, the CM protection decreased when CM was obtained from higher-passage MSCs and increasing MSC donor age. In addition, the neuroprotective effect was lost when MSCs were isolated from MS patients [114].

## 8. Conclusions

MSCs seem promising in MS treatment due to their immunomodulatory capacity, release of trophic factors, differentiation ability, and regenerative potential. Experimental results suggested that MSCs from different sources, and transplanted through different routes, decreased demyelination and inflammatory cell infiltration leading to an amelioration of clinical scores and symptoms. In addition, preconditioned and differentiated MSCs, but also MSCs in combination with different compounds, showed improved therapeutic potential compared to native MSCs and exerted better protective effects in MS models. However, a limit of experimental studies is the use of animals of a single gender, given that sex-dependent differences exist. Moreover, the use of different MS models, different number of transplanted cells, different MSCs sources, and routes of administration make difficult to define the optimal treatment in terms of cell type, dose, and administration conditions. Also, the secretome of MSCs was able to exert protective actions in MS experimental models and then, taking into consideration the reported advantages, it may represent a better approach compared to MSCs. However, it is important to administer MSCs or the secretome at the onset or at least in a precocious phase of the disease. Clinical trials evidenced the safety and feasibility of MSCs treatment, and also some improvements, but more data on larger cohorts are required to establish their efficacy. Moreover, it needs to be confirmed if autologous MSCs therapy is possible in MS patients given that there are controversial results about the features of MSCs derived from MS patients.

## Figures and Tables

**Table 1:** Overview of the experiments involving the use of MSCs in MS experimental models.

Age and Strain	MS Model	MSCs	MSCs Administration	Results	Ref.
4-week-old female C57BL/6 mice	MOG-EAE	Mouse BM-MSCs	1 × 10^7^ cells; i.p. at the disease onset (day 14) and day 20	↑ clinical score; ↓ inflammatory infiltration and demyelination of spinal cord	[23]
Adult male Swiss mice	Spinal cord homogenate EAE	Rat BM-MSCs	1 × 10^6^; i.p. on the same day of immunization	↑ clinical score; ↓ inflammation	[24]
10–14-week-old female C57BL/6 mice	MOG-EAE	Mouse BM-MSCs	1 × 10^6^ cells; i.p. 5 days after EAE induction	IL17/IL17RA signaling mediated the therapeutic function of MSCs in EAE	[25]
6–8-week-old female C57BL/6 mice	MOG-EAE	BM-MSCs	5 × 10^5^ cells; i.v. at the disease onset (day 11)	↑ neurobehavioral outcomes; ↓ BBB disruption, inflammatory infiltration and demyelination in spinal cord	[26]
6–8-week-old female C57BL/6J mice	MOG-EAE	Mouse BM-MSCs	1 × 10^6^; i.v. on the day of clinical onset	↓ vascular alteration of vessels, myelin, and neuronal damage	[27]
2-month-old female C57BL6/J mice	MOG-EAE	Human BM-MSCs	1 × 10^6^; i.v. 7 days after EAE induction	↑ retinal ganglion cell function and motor-sensory impairment	[28]
10–14-week-old females C57BL/6 mice	MOG-EAE	Mouse BM-MSCs	1 × 10^6^; i.v. administrated at the onset of the disease (day 10), at the peak of the disease (day 18), or at the time of EAE stabilization (day 30)	↑ clinical score; ↓ Th1, Th17, and Treg	[29]
Female C57BL/6 mice	MOG-EAE and cuprizone model	Mouse BM-MSCs	5 × 10^6^ cells; EAE: i.p. on day 3 and 8. Cuprizone: i.p. weekly	↓ Th17 activation and function; positive effects only in cuprizone model	[30]
6-week-old male C57BL/6 mice	Cuprizone model	Mouse BM-MSCs	3 × 10^5^ cells; lateral ventricle after cuprizone treatment	↑ M2 phenotype; ↓ M1 phenotype	[31]
8-week-old male C57BL/6 mice	Cuprizone model	Mouse BM-MSCs	3 × 10^5^ cells; lateral ventricle after 12 weeks of diet with cuprizone	↑ remyelination; ↓ microgliosis and astrocytosis	[32]
7–8-week-old male C57BL/6 mice	Cuprizone model	Human BM-MSCs	2 × 10^6^ cells for 2 consecutive weeks i.p. after 4 weeks of cuprizone treatment	↓ demyelination in corpus callosum	[33]
7–8-week-old male C57BL/6 mice	Cuprizone model	Mouse BM-MSCs	1 × 10^6^; i.v. 2 weeks after cuprizone diet	↑ remyelination; ↓ demyelination and apoptosis	[34]
12-month-old male Fischer 344 rats	Ethidium bromide	Rat BM-MSCs (2-month-old and 17–20-month-old rat donors)	1.5–2.0 × 10^6^ MSCs; i.v. at 1, 2, and 3 days post-lesion induction	↑ differentiation of OPC with young MSCs	[35]
6–8-week-old female C57BL/6 mice	MOG-EAE	Mouse AD-MSCs expanded in hypoxia or human AD-MSCs	1 × 10^6^ cells; i.p. at the disease onset or at the acute phase	↓ disease severity, inflammatory cell infiltration, and demyelination	[36]
Dogs	Demyelinating leukoencephalitis caused by the distemper virus	Canine AD-MSCs	1 × 10^7^; 3 doses into the femoral artery at 30-day intervals	↓ myoclonus	[37]
6–8-week-old female C57Bl/6 mice	MOG-EAE	Human AD-MSCs	1 × 10^6^; i.p. before disease onset or at the peak of disease severity	Obese derived AD-MSCs failed to improve EAE	[38]
6–8-week-old female C57Bl/6 mice	MOG-EAE	Mouse AD-MSCs	1 × 10^6^; i.p. 20 days after immunization	↑ clinical score, behavior, motor function, and histopathologic analyses	[39]
6–8-week-old female C57Bl/6 mice	MOG-EAE	Mouse AD-MSCs	1 × 10^6^; i.p. 8 days after immunization	↑ SVF cells and AD-MSCs administration transiently increased disease severity. SVF cells were able to overcome the advancing pathogenesis and showed improvements	[40]
12-week-old male C57BL/6 mice	MOG-EAE	Human PDLSCs	1 × 10^6^ cells; i.v. at the disease onset (day 14)	↑ clinical score, lymphocytic infiltration and demyelination; ↓ apoptosis	[41]
6–8-week-old C57BL/6J mice	MOG-EAE	Mouse S-MSCs	1 × 10^6^; i.p. 3 days before immunization or on day 8 post immunization	↑ expression of Th2- and Treg-associated genes; ↓ disease onset, disease severity, demyelination, inflammatory cell infiltrate, Th17 cell induction	[42]
8-week-old male C57BL/6 mice	Cuprizone model	Human WJ-MSCs	3 × 10^5^ cells; lateral ventricle after 12 weeks of diet with cuprizone	↑ remyelination and oligodendrocyte; ↓ astrogliosis, microgliosis, oxidative stress, and mitochondrial dysfunction	[43]
8-week-old female dark agouti rats	MOG-EAE	Human WJ-MSCs	2 × 10^6^; i.v. at the onset of clinical symptoms. In order to assess the effect on chronic disease course, rats received a dose at 28 days post immunization	↑ improved clinical score in EAE rats; ↓ proliferation of activated T	[44]
3–5-year-old cynomolgus monkeys	MOG-EAE	Human UCMSCs	1 × 10^6^ cells/kg/mL; i.v. at days 74 and 84	↓ demyelination and inflammation	[45]
8–10-week-old female C57BL/6 mice	MOG-EAE	Human AMCs	1 × 10^6^ cells; i.p. at the disease onset (day 14)	↑ remyelination and neurotrophic factors; ↓ clinical score, inflammation	[46]
6–8-week-old female Wistar rats	MOG-EAE	Human PMSCs	1 × 10^6^ cells; i.v. at 9 or 16 days post immunization	↑ oligodendrocyte precursors; ↓ inflammation, axonal injury and degenerating neurons	[47]
9–10-week-old male Lewis rats	Spinal cord homogenate EAE	Rat PMSCs; EMSCs	1 × 10^6^ cells; ICV 10 days after EAE induction	↑ neurological functions; ↓ infiltrating inflammatory cells, gliosis, apoptosis, and demyelination	[48]
8–9-year-old female cynomolgus monkeys	MOG-EAE	EMSCs	2 × 10^7^ cells/monkey; i.t. 3 doses	↓ clinical symptoms, brain lesion, and demyelination	[49]
10–14-week-old female C57BL/6 mice	MOG-EAE	Human DMSCs	1 × 10^6^ cells; i.p. at days 1, 3 and 6, or days 6-10 after MOG inoculation.	↓ demyelination and inflammatory cell infiltration	[50]
Female C57BL/6J mice	MOG-EAE	Mouse BM-MSCs	1 × 10^6^ cells; i.v. at the disease onset (day 11) or at day 3 and 8	No improvements in EAE	[51]
10-week-old male C57BL/6J mice	Cuprizone model	Human and murine BM-MSCs; canine AD-MSCs	1 × 10^6^ cells; intraventricularly or intralesion at week 3 or week 4	No regenerative effects	[52]
12-week-old male C57BL/6 mice	MOG-EAE	Mouse BM-MSCs	1 × 10^6^; i.v. 8 days after immunization	Superior effects of mMAPCs compared to MSCs	[53]
10–12-week-old female C57BL/6 mice	MOG-EAE	EAE or naïve mouse BM-MSCs; human BM-MSCs obtained from RRMS or control subjects	0.8 × 10^6^; i.v. 15 days post immunization	BM-MSCs obtained from EAE mice did not improve EAE	[54]

AD-MSCs, Adipose tissue-derived MSCs; AMC, amnion mesenchymal cells; BBB, blood-brain barrier; BM-MSCs, Bone marrow MSCs; DMSCs, Decidua derived MSCs; EAE, experimental autoimmune encephalomyelitis; EMCSs, Embryonic MSCs; IL, interleukin; i.p. intraperitoneal; i.v. intravenous; IL17RA, IL17 Receptor A; mMAPCs, Mouse multipotent adult progenitor cells; MOG, myelin oligodendrocyte glycoprotein; MSCs, mesenchymal stem cells; OPC, Oligodendrocyte progenitor cell; PMSCs, Placental derived MSCs; SVF, Stromal vascular fraction; Th, T helper; Treg, regulatory T; UCMSCs, Umbilical cord MSCs; WJ-MSCs, Wharton’s jelly-derived MSCs. ↑, enhancement; ↓, reduction.

**Table 2:** Overview of the experiments involving the use of differentiated MSCs in MS experimental models.

Age and Strain	MS Model	MSCs	Differentiation Method	MSCs Administration	Results	Ref.
6–7-week-old female C57BL/6 mice	MOG-EAE	Human and mouse BM-MSCs	DMEM-F12 supplemented with B-27, bFGF, EGF for 14 days	Intraventricularly 8 days after EAE induction	↑ disease score; ↓ inflammation, axonal loss, and demyelination	[60]
12-week-old female C57BL/6 mice	MOG-EAE	Mouse BM-MSCs	Neurobasal media supplemented with B27, insulin-transferring-selenite, L-glutamine, penicillin and streptomycin, bFGF and dehydroepiandrosterone	1 × 10^6^; i.v. on days 22, 29, and 36 after immunization	↑ IL-10; ↓ proliferation of pathogenic MOG35-55-specific T cells, IFN-γ production	[61]
8–10-week-old female C57BL/6 mice	MOG-EAE	Human UCB-MSCs	Basal medium supplemented with B-27, L-glutamin, retinoic acid, bFGF, epidermal growth factor, nerve growth factor, 3-isobutylmethyl-xanthin, and ascorbic acid	5 × 10^5^ UCB-MSCs or MDNPC; i.v. on days 14 and 21 after immunization	↑ clinical score; ↓ leukocyte infiltration	[62]
Female Sprague-Dawley rats	LPC	Rat BM-MSCs	DMEM/F12 with N-2 supplement, B27 supplement, bFGF and epidermal growth factor for 8–12 days	2 × 10^5^; injected into the corpus callosum	↓ demyelination	[63]

bFGF, basic fibroblast growth factor; BM-MSCs, Bone marrow MSCs; EAE, experimental autoimmune encephalomyelitis; IL, interleukin; IFN, Interferon; i.v., intravenous; MOG, myelin oligodendrocyte glycoprotein; MSCs, mesenchymal stem cells; LPC, lysophosphatidylcholine; UCB-MSCs, Umbilical cord blood mesenchymal stromal cells. ↑, enhancement; ↓, reduction.

**Table 3:** Overview of the experiments involving the use of preconditioned MSCs in MS experimental models.

Age and Strain	MS Model	MSCs	Pre-Conditioning	MSCs Administration	Results	Ref.
6–8-week-old female C57BL/6J mice	MOG-EAE	Human UCMSCs	20 ng/mL IFN-γ for 48 h	1 × 10^6^; i.v. 14 days after immunization	↑ clinical symptoms; ↓ serum IL-17A and TNF-α levels	[64]
10-week-old female C57BL/6 mice	MOG-EAE	Human UCMSCs	TMP 100 µM	1 × 10^6^; i.v. 13 days after immunization	↑ clinical score; ↓ inflammatory cell infiltration and NLRP3 levels, demyelination, and BBB disruption	[65]
6–8-week-old male C57BL/6 mice	Cuprizone	Mouse BM-MSCs	100 ng/mL SDF-1α for 24 h	1 × 10^6^; intranasal	↑ remyelination	[66]
6-8-week-old male Wistar rats	Spinal cord homogenate EAE	Rat BM-MSCs	100 nM 17β-estradiol for 24 h	2 × 10^6^ cells/rat; i.p.	↑ clinical score and neuropathological changes	[67]
8–12-week-old male Wistar rats	Spinal cord homogenate EAE	Rat BM-MSCs	100 nM 17β-estradiol for 24 h	2 × 10^6^ cells/rat; i.p. when all of the rats showed disease symptoms	↓ lymphocyte infiltration	[68]
2–5-year old and of both sexes mongrel dogs	Ethidium bromide	Dog SVF	low level laser for 20 min	10 × 10^6^ nucleated cells; injected directly in the Cerebrospinal fluid 14 days after induction	↑ remyelination	[69]

BM-MSCs, Bone marrow MSCs; EAE, experimental autoimmune encephalomyelitis; IFN, Interferon; IL, interleukin; i.p., intraperitoneal; i.v., intravenous; MOG, myelin oligodendrocyte glycoprotein; MSCs, mesenchymal stem cells; SDF-1α, stromal cell-derived factor-1; SVF, Stromal vascular fraction; TMP, Tetramethylpyrazine; TNF, tumor necrosis factor. ↑, enhancement; ↓, reduction.

**Table 4:** Overview of the experiments involving the use of engineered MSCs in MS experimental models.

Age and Strain	MS Model	MSCs	Gene Expression	MSCs Administration	Results	Ref.
6–8-week-old female C57Bl/6 mice	MOG-EAE	Mouse AD-MSCs	Mouse IFN-β gene	5 × 10^5^/each time; i.v. on day 16 and 18 after immunization	↑ Tregs and IL-10 production; ↓ inflammatory cell infiltration	[70]
6–8-week-old female SJL/JCrl (RR-EAE) and C57Bl/6 mice (CP-EAE)	Proteolipid protein 139–151 peptide for RR-EAE, MOG for CP-EAE	Mouse AD-MSCs	Mouse IFN-β gene	1 × 10^6^; i.v.	↓ disease score, activated microglia and inflammatory cell infiltration and demyelination	[71]
C57BL/6 mice	MOG-EAE	Human BM-MSCs	PSGL-1, FUT-7 and IL-10	1 × 10^6^; i.v. 14 days after immunization	↑ clinical score and myelination; ↓ inflammatory infiltration	[72]
6–8-week-old female C57BL/6 J mice	MOG-EAE	Human UCMSCs	SPK1	5.5 × 10^6^; i.v. on the day 7, 14, 21, 28	↓ neurological deficits, inflammatory cell infiltration, demyelination, and astrogliosis	[73]

BM-MSCs, Bone marrow MSCs; EAE, experimental autoimmune encephalomyelitis; MOG, myelin oligodendrocyte glycoprotein; IFN, Interferon; IL—interleukin; i.v., intravenous; MSCs, mesenchymal stem cells; PSGL1, P-selectin glycoprotein ligand-1; Treg, Regulatory T; UCMSCs, Umbilical cord MSCs. ↑, enhancement; ↓, reduction.

**Table 5:** Overview of the experiments involving the use of MSC secretome in MS experimental models.

Age and Strain	MS Model	MSCs Source	Secretome	Administration	Results	Ref.
Female C57BL/6 mice	MOG-EAE	Mouse BM-MSCs	CM	60 μL/mouse (30 μL on each nostril); i.n. from the day 3 after immunization until the onset of symptoms	↓ disease onset and disease severity demyelination B cells infiltration and microglia activation	[74]
8-week-old female C57BL/6J mice	MOG-EAE	SHED	CM	500 µl; i.v. 14 days after immunization corresponding to the peak of EAE	↑ clinical scores; ↓ demyelination, axonal injury, inflammatory infiltrates, and the expression of proinflammatory cytokines	[75]
6–7-week-old female C57BL/6 mice	MOG-EAE	Mouse AD-MSCs	CM	1 mL; i.p. on day 10 after immunization and the same amount once a week for four weeks. 1 × 10^6^ AD-MSCs i.p.	↑ clinical scores; ↓ inflammatory infiltration	[76]
6–7-week-old female C57BL/6 mice	MOG-EAE	Human PDLSCs	CM obtained in hypoxic condition	1.0 mg/mouse; i.v. after 14 days from EAE induction	↑ IL-37 and BDNF levels; ↓ inflammatory cell infiltration, demyelination, oxidative stress, and apoptosis	[77]
12-week-old male C57BL/6 mice	MOG-EAE	Human PDLSCs derived from RRMS patients and healthy controls	CM and EMVs	1600 μG for CM or 24 μG for EMVs; i.v. after 14 days of EAE induction	↓ inflammasome, NF-κB and inflammatory cell infiltration	[78]
10-week-old male C57BL/6 mice	MOG-EAE	Human WJ-MSCs differentiated toward oligodendrocytes	CM	i.n. 10 days after immunization	↑ remyelination and neurological scores; ↓ inflammatory cell infiltration and the expression of proinflammatory markers	[79]
4–6-week-old female SJL/J mice	TMEV-induced demyelinating disease	Human AD-MSCs	EVs	25 μg; i.v. on day 60 post infection	↑ motor function; ↓ brain atrophy, inflammatory infiltration and plasma cytokine levels	[80]
6–8-week-old female C57Bl/6 mice	MOG-EAE	Human AD-MSCs	EVs	60 μg EVs or 1 × 10^6^ AD-MSC; i.v. 10 days after immunization	↑ clinical score; ↓ inflammatory infiltrates and demyelination	[81]
3-month-old male and female C57BL/6J mice	MOG-EAE	Human PMSCs	EVs	1 × 10^7^ (low dose), 1 × 10^10^ (high dose) EVs, 1 × 10^6^ PMSCs; i.v. on day 19 (peak of the disease)	↑ motor function, myelination	[82]
6–8-week-old female C57BL/6J mice	MOG-EAE	Human BM-MSCs	Exo	150 μg or 1 × 10^6^ native or IFN-γ stimulated MSCs; i.v. at the peak of the disease (day 18)	↑ Tregs; ↓ demyelination and neuroinflammation	[83]
Female Sprague Dawley rats	Spinal cord homogenate EAE	Rat BM-MSCs	Exo	100 μg or 400 µg; i.v. the day after EAE induction. 1 × 10^6^ BM-MSCs	↑ M2-related markers and behavioral scores; ↓ demyelination and inflammatory cell infiltration	[84]
10–13-week-old female C57BL/6 mice	MOG-EAE	Mouse BM-MSCs	Exo	200 μg of Exo or Exo-APT; i.v. on day 1, 3, 6 after immunization in the prophylactic model or on day 12, 15, and 18 in the therapeutic model	↓ disease severity, inflammation, and demyelination	[85]

AD-MSCs, Adipose tissue-derived MSCs; BM-MSCs, Bone marrow MSCs; CM, conditioned medium; EAE, experimental autoimmune encephalomyelitis; EVs, Extracellular vesicles; Exo, exosomes; IFN, interferon; i.p., intraperitoneal; i.v., intravenous; MOG, myelin oligodendrocyte glycoprotein; MSCs, mesenchymal stem cells; PDLSCs, Periodontal Ligament Stem Cells; PMSCs, Placental derived MSCs; RRMS, relapsing remitting multiple sclerosis; SHED, stem cells from human exfoliated deciduous teeth; TMEV, Theiler’s murine encephalomyelitis virus; Treg, Regulatory T. ↑, enhancement; ↓, reduction.

**Table 6:** Overview of the experiments involving the use of MSCs in combination with other therapies in MS experimental models.

Age and Strain	MS Model	MSCs	Combination	MSCs Administration	Results	Ref.
9-week-old female C57BL/6 mice	MOG-EAE	Human BM-MSCs	MP (20 mg/kg) and BM-MSCs	1 × 10^6^; i.v. (MSCs) or i.p. (MP)14 days after immunization	↑ remyelination; ↓ inflammatory cell infiltration	[86]
9-week-old female C57BL/6 mice	MOG-EAE	Human BM-MSCs	MP (10 mg/kg) and MSCs-IFNβ	1 × 10^6^; i.v. (MSCs) or i.p. (MP) 14 days after immunization	↑ remyelination; ↓ inflammatory infiltration, BBB disruption	[87]
8–12-week-old female C57BL/6 mice	MOG-EAE	Mouse BM-MSCs	Resveratrol (i.p. 30 mg/kg) and BM-MSCs	1.5×10^6^ cells; i.v. 7 days after immunization	↓ symptom onset, clinical scores, and inflammatory cell infiltration	[88]
6–8-week-old male Wistar rats	Spinal cord homogenate EAE	Rat BM-MSCs	Nicotine (i.p. 2.5 mg/kg body weight every day) and BM-MSCs	2 × 10^6^; i.p. 12 days after immunization	↓ disease disability	[89]
8–10-week-old female C57BL/6 mice	MOG-EAE	Mouse BM-MSCs	Rapamycin (0.3 mg/kg) and BM-MSCs	2 × 10^6^; i.p. 10 and 17 days after EAE induction	↑ clinical score; ↓ inflammatory cell infiltration and demyelination	[90]
8–12-week-old female C57BL/6 mice	MOG-EAE	Mouse BM-MSCs	Fasudil (i.p. 400 μg/mice on day 14 p.i. till day 27) and BM-MSCs	1.5 × 10^5^; intranasally on day 12 post immunization	↑ clinical score, demyelination, and inflammatory cell infiltration	[91]
Female SD rats	Ethidium bromide	Rat BM-MSCs	NT-3 (20 ng/mL) and/or RA (1 μM/L) preinduced MSCs and electroacupuncture	1 × 10^5^; intralesion	↑ conduction of cortical motor-evoked potentials; ↓ demyelination	[92]
8-week-old male C57BL/6 mice	Cuprizone	Mouse BM-MSCs	PLX3397 (290 mg/kg) for 21 days and BM-MSCs	3 × 10^5^ cells; intraventricular at the 13th week	↑ oligodendrocytes and remyelination; ↓ microglia, astrocytes, and neurobehavioral deficits	[93]

BM-MSCs, Bone marrow MSCs; EAE, experimental autoimmune encephalomyelitis; i.p., intraperitoneal; i.v., intravenous; MP, methylprednisolone; MOG, myelin oligodendrocyte glycoprotein; MSCs, mesenchymal stem cells; NT-3, neurotrophin-3; RA, retinoic acid. ↑, enhancement; ↓, reduction.

**Table 7:** Overview of the clinical trials registered on Clinicaltrial.gov involving the use of MSCs in MS patients.

NCT Number	MSCs	MSCs Administration	Safety	Severe Adverse Events	Improvements	Ref.
NCT00813969	Autologous BM-MSCs	1–2 × 10^6^/Kg body weight; i.v.	Yes	No	-	[95]
NCT01895439	Autologous BM-MSCs and CM	Average number of 110 × 10^6^ MSCs injected per patient; intrathecal. A month after, an average volume of 18 mL of CM was given intrathecally	Yes	No	Yes	[97]
NCT01056471	Autologous AD-MSCs	1 × 10^6^ cells/kg (low-dose) or 4 × 10^6^ cells/kg (high-dose); i.v.	Yes	No	-	[98]
NCT02034188	UCMSC	20 × 10^6^/day; i.v. over the course of 7 visit separated by 1–4 days	Yes	No	Yes	[99]
NCT01364246	UCMSCs	Four doses: 4 × 10^7^, 2 × 10^7^, 2 × 10^7^, 2 × 10^7^ i.v. on day 0, 7, 14, and 21. Three doses: 2 × 10^7^, 2 × 10^7^, 2 × 10^7^ intrathecally on day 7, 14, and 21	Yes	No	-	[100]
NCT01933802	Neural progenitors derived from autologous BM-MSCs	Up to 1 × 10^7^; intrathecal, 3 doses spaced three months apart	Yes	No	Yes	[102]

AD-MSCs, adipose tissue-derived MSCs; BM-MSCs, bone marrow MSCs; CM, conditioned medium; i.v., intravenous; MSCs, mesenchymal stem cells; UCMSCs, Umbilical cord MSCs.

**Table 8:** Overview of the clinical trials involving the use of MSCs in MS patients.

MSCs	MSCs Administration	Severe Adverse Events	Improvements	Ref.
Autologous BM-MSCs	A mean of 57 × 10^6^ cells; intrathecally	No	Yes	[105]
UCMSC	1 to 2 × 10^6^ cells/kg; i.v. at 3-month intervals for 7 times	No	Yes	[106]
Autologous SVF	Patients received intraventricular SVF injection volumes of 3.5–20 cc (median: 4 cc) containing 4.05 × 10^5^ to 6.2 × 10^7^ cells/cc and contained on average 8% hematopoietic and 7.5% ADSCs	-	Yes	[107]
SVF	4.2 × 10^6^ cells (females) and 12.8 × 10^6^ cells (males); intrathecally	No	-	[108]

BM-MSCs, bone marrow MSCs; SVF, Stromal vascular fraction; UCMSCs, Umbilical cord MSCs.

## Abbreviations

MS	Multiple sclerosis
CNS	Central nervous system
CIS	Clinically isolated syndrome
RRMS	Relapsing remitting MS
SPMS	Secondary progressive MS
PPMS	Primary progressive MS
Th	T helper
MSCs	Mesenchymal stem cells
CM	conditioned medium
EVs	Extracellular vesicles
Exo	Exosomes
OPC	Oligodendrocyte progenitor cell
Olig2	Oligodendrocyte transcription factor
MBP	Myelin basic protein
GFAP	Glial fibrillary acidic protein
Treg	Regulatory T
BM-MSCs	Bone marrow MSCs
EAE	Experimental autoimmune encephalomyelitis
TNF	Tumor necrosis factor
MPO	Myeloperoxidase
NO	Nitric oxide
IL	Interleukin
GSH	Glutathione
MP	Methylprednisolone
IL17RA	IL17 Receptor A
BBB	Blood-brain barrier
AQP4	Aquaporin 4
A2BAR	A2B adenosine receptor
HIF-1	Hypoxia-inducible factor 1
T-bet	T-box transcription factor TBX21
RORγT	RAR-related orphan receptor γ
Foxp3	Forkhead box P3
TGF	Transforming growth factor
AD-MSCs	Adipose tissue-derived MSCs
DC	Dendritic cell
SVF	Stromal vascular fraction
PDLSCs	Periodontal Ligament Stem Cells
NGF	Nerve growth factor
BDNF	Brain derived neurotrophic factor
S-MSCs	Skin-derived MSCs
IFN	Interferon
sTNFR1	soluble TNF receptor 1
WJ-MSCs	Wharton’s jelly-derived MSCs
MDA	Malondialdehyde
SOD	Superoxide dismutase
IDO1	indoleamine 2,3-dioxygenase 1
UCMSCs	Umbilical cord MSCs
NK	Natural killer
AMCs	Amnion mesenchymal cells
PMSCs	Placental derived MSCs
EMSCs	Embryonic MSCs
DMSCs	Decidua derived MSCs
mMAPCs	Mouse multipotent adult progenitor cells
NT-3	Neurotrophin-3
NMSCs	Neutralized MSCs
MSCs-NPs	MSCs-derived neural progenitor
MOG	Myelin oligodendrocyte glycoprotein
UCB-MSC	Umbilical cord blood mesenchymal stromal cells
TMP	Tetramethylpyrazine
SDF-1α	stromal cell-derived factor-1α
CXCR4	C-X-C chemokinereceptor type 4
APC	Adenomatous polyposis coli
17β-ED	17β-estradiol
MMP	Metalloproteinase
PSGL1	P-selectin glycoprotein ligand-1
SLeX	Sialyl-Lewisx
SPK1	Sphingosine kinase 1
rMOG	recombinant MOG
SHED	human exfoliated deciduous teeth
ED–Siglec-9	secreted ectodomain of sialic acid–binding Ig-like lectin-9
HGF	Hepatocyte growth factor
H-PDLSCs-CM	CM obtained from human PDLSCs under hypoxia
EMVs	Exo/microvesicles
TMEV	Theiler’s murine encephalomyelitis virus
GDNF	glial cell-derived neurotrophic factor
CNTF	ciliary neurotrophic factor
RA	retinoic acid
NR-MSCs	preinduced MSCs with NT-3 and RA
EA	Electroacupuncture
EDSS	Expanded Disability Status Scale
MRI	Magnetic resonance imaging
RD	radial diffusivity
AD	axial diffusivity
